# Dodecatungstophosphoric Acid (H_3_PW_12_O_40_), Samarium and Ruthenium (ІІІ) Chloride Catalyzed Synthesis of Unsaturated 2-Phenyl-5(4H)-oxazolone Derivatives under Solvent-free Conditions

**DOI:** 10.3390/molecules13123246

**Published:** 2008-12-18

**Authors:** Ahmad Momeni Tikdari, Samieh Fozooni, Hooshang Hamidian

**Affiliations:** 1 Department of Chemistry, Shahid Bahonar University of Kerman, Kerman, 76135-133, Iran; 2 Department of Chemistry, Payame Noor University (PNU), Kerman, Iran

**Keywords:** 2-Phenyl-5(4H)-oxazolone, Microwave irradiation, Samarium, Ruthenium(ΙΙΙ) chloride, Dodecatungstophosphoric acid.

## Abstract

We have found that dodecatungstophosphoric acid (H_3_PW_12_O_40_), samarium or ruthenium(ΙΙΙ) chloride act as efficient catalysts for the synthesis of unsaturated 2-phenyl-5(4H)oxazolone derivatives under solvent-free conditions. The key features of the reported protocols are short reaction times, high yields of products under ambient conditions and simple workups.

## Introduction

Heterocyclic compounds have acquired more importance in recent years due to their pharmacological activities [1, 2] and nitrogen-, sulphur- and oxygen-containing five/six member heterocyclic compounds have achieved enormous significance in the field of Medicinal Chemistry. Oxazole plays a very pivotal role in the manufacture of various biologically active drugs with analgesic, anti-inflammatory, anti-depressant, anti-cancer, anti-microbial, anti-diabetic and antiobesity properties [3,4,5].

2-Phenyl-5(4H)-oxazolones are important intermediates in the synthesis of several molecules including amino acids, peptides, antimicrobial or antitumor compounds and heterocyclic precursors, as well as in biosensor coupling and photosensitive composition devices for proteins [6,7,8,9,10,11].

A number of methods are available for the synthesis of azalactones, including the use of acetic anhydride and sodium acetate, lead acetate, polyphosphoric acid, sulphur trioxide/dimethylformamide complex, perchloric acid and carbodiimides [12,13,14,15]. Recently, synthesis of oxazolones using anhydrous zinc chloride or bismuth (ΙΙΙ) acetate as catalysts has been reported [15,16,17,18,19]. 

Microwave irradiation is a nonconventional energy source whose popularity and synthetic utility in Organic Chemistry has increased considerably in recent years. The rapid heating induced by such radiation avoids harsh classical conditions and the decomposition of the reagents, leading to the formation of products under mild reaction conditions, thus typically increasing the yields. The elimination of toxic organic solvents and use of catalysts is one of the most important goals in Green Chemistry. Coupling of these two techniques, that is, organic reactions using catalyst with microwave irradiation has been a field that has shown excellent results leading to the development of many reaction procedures, which are environmental friendly, thus falling within the domain of Green Chemistry [20, 21]. 

We report herein that dodecatungstophosphoric acid (H_3_PW_12_O_40_), Sm or RuCl_3_ act as efficient heterogeneous catalysts for the synthesis of 2-phenyl-5(4H)-oxazolone derivatives. In recent years, the use of solid acids as heterogeneous catalysts has received considerable attention in different areas of organic synthesis. Heteropolyacids (HPAs) are attractive, because of their flexibility in modifying the acid strength, environmental compatibility, no toxicity and experimental simplicity. The use of HPA as a catalyst makes the process convenient and environmentally benign [22,23,24].

There have been extensive efforts to utilize the potential of the HPAs in synthetic Organic Chemistry such as deprotection of *t*-butyldimethylsilanes, regioselective aerobic oxygenation of nitrobenzene to 2-nitrophenol and oxidation of aliphatic, benzylic and allylic alcohols using dimethyl sulfoxides as oxygen transfer agents. Heteropolyacids, due to their unique physicochemical properties are widely used in variety of acid catalyzed reactions, such as esterification, hydration and dehydration, de-esterification, polymerization, condensation in homogenous and heterogenous systems [25,26,27].

The direct use of metallic samarium in organic transformations has attracted the attention of many organic chemists. In most cases, the reactions promoted by samarium were carried out in THF, and metallic samarium had to be activated or pretreated by various methods so as to ensure the reactions proceed smoothly. Only a few reports could be found in the literature concerning organic reactions promoted efficiently by metallic samarium without activation or pretreatment. We now report a novel and facile synthesis of 2-phenyl-5(4H)oxazolone compounds promoted by metallic samarium [28, 29]. 

Ruthenium(ΙΙΙ) trichloride has been widely used to catalyze oxidations, reductions or polymerizations of organic compounds. Ruthenium compounds are advantageous as they can be used both in acidic as well as in alkaline medium [30,31].

## Results and Discussion

In the past, the synthesis of unsaturated 2-phenyl-5(4H)-oxazolones have been accomplished by the classical Erlenmeyer reaction, involving condensation of hippuric acid, aldehydes or ketones in presence of acetic anhydride and sodium acetate under refluxing conditions. This procedure requires long reaction times and the yields are moderate. We report here three solvent-free procedures for the synthesis of unsaturated 2-phenyl-5(4H)-oxazolones **3a-j** from appropriate aldehydes or ketones (**1**) with hippuric acid (**2**) under microwave irradiation in the presence of three different catalysts, namely dodecatungstophosphoric acid, samarium and ruthenium(ΙΙΙ) chloride. Then we contemplated a comparison between dodecatungstophosphoric acid, samarium and ruthenium(ΙΙΙ) chloride, which have not been employed for the synthesis of 2-phenyl-5(4H)-oxazolones, in terms of the yield and rate of the reaction.

**Scheme 1:**
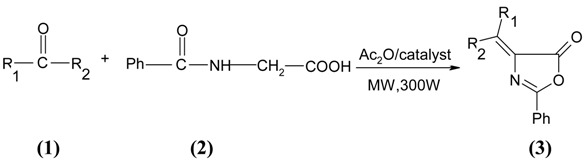
Synthesis of 5(4H)-oxazolones in the presence of H_3_PW_12_O_40_, Sm, RuCl_3__._

A reaction mixture consisting of hippuric acid, aldehydes or ketones, in the presence of acetic anhydride and corresponding catalysts, was exposed to microwaves for a suitable time (Table 1). The mixture turned solid at room temperature and led to the isolation of 2-phenyl-5(4H)-oxazolone derivatives (Scheme 1). It was evidently observed that the yield and rate of the reaction were high in all the cases. Dodecatungstophosphoric acid, samarium and ruthenium(ΙΙΙ) chloride appeared to be efficient catalysts. The comparisons between these catalysts are shown in Table 1.

Catalysts: H_3_PW_12_O_40_, Sm, RuC_l3_

**Table:** 

**1a)** Benzaldehyde	**1f)** 4-(*N,N*-Dimethylamino)benzaldehyde
**1b)** 4-Chlorobenzaldehyde	**1g)** 4-Methylbenzaldehyde
**1c)** 2,4-Dichlorobenzaldehyde	**1h)** Furfural
**1d)** 3-Nitrobenzaldehyde	**1i)** Thiophenecarboxaldehyde
**1e)** 4-Methoxybenzaldehyde	**1j)** Cyclohexanone

It was observed that the reaction proceeds faster in the case of dodecatungstophosphoric acid, but the products were obtained in high yields in the case of samarium. The structures of the products were confirmed by IR, ^1^H-NMR and mass spectroscopy (Table 2).

In conclusion, we have reported novel, simple, efficient and modified methods for synthesis of unsaturated 2-phenyl-5(4H)-oxazolone derivatives. The reaction is catalyzed by dodecatungsto-phosphoric acid (H_3_PW_12_O_40_), samarium and ruthenium(ΙΙΙ) chloride, which are environmentally benign.

Yields of the products, short reaction times, easing of work-up and preclusion of toxic solvents are the striking features of the present protocol.

**Table 1:** Comparison of H_3_PW_12_O_40_, RuCl_3_ and Sm as catalysts for synthesis of 2-phenyl-5(4H)-oxazolones in terms of yield and time.

Product	Y(%)(t)^a^	Y(%)(t)^b^	Y(%)(t)^c^	m.p.(◦C)	m.p.(Lit)
**3a**	87(2)	80(3)	92(5)	160-161	158 [13]
**3b**	83(2.5)	73(4)	87(7)	189-190	185 [14]
**3c**	93(3)	86(5)	96(8)	181-182	183 [14]
**3d**	87(3)	82(5)	93(7)	174-175	175-176 [15]
**3e**	95(1.5)	87(2)	97(4)	158-159	159 [16]
**3f**	92(1.5)	87(2)	95(4)	212-213	213-214 [15]
**3g**	89(2)	82(3)	93(5)	146-147	145-146 [18]
**3h**	84(2)	80(3)	87(5)	171-172	171[16]
**3i**	89(2.5)	84(4)	93(6)	178-179	180 [16]
**3j**	77(2)	71(3)	82(4)	138-139	137-138 [15]

^a^Yield(time/min) using H_3_PW_12_O_40_ ^b ^Yield(time/min) using RuCl_3_ ^c ^Yield(time/min) using Sm

**Table 2:** Spectral Data of Compounds **3a-j**.

Product	IR (KBr)/υ [cm^-1^]	^1^H-NMR (CDCl_3_)/δ[ppm]	MS/m/z (%)
**3a**	1790, 1650	7.24 (s, 1H, vinyl); 7.44-8.12 (m, 10H, ArH)	249 (M^+^, 15), 105 (100), 77 (70)
**3b**	1800,1650	7.44-8.12 (m, 10H, vinyl and ArH)	285 (M+2), 283 (M^+^, 15), 105 (100), 77 (70)
**3c**	1800, 1660	7.36-8.08 (m, 9H, vinyl and ArH)	319 (M+4, 2), 317 (M+2, 8), 318 (M^+^, 10), 105 (100), 77 (70)
**3d**	1800,1660	7.42-8.29 (m, 10H, vinyl and ArH)	294 (M^+^, 15), 105 (100), 77 (70)
**3e**	1790,1660	4.02 (s, 3H, CH_3_); 7.07 (s, 1H, vinyl); 7.25-8.22 (m, 9H, ArH)	279 (M^+^, 10), 105 (100), 77 (70)
**3f**	1810, 1660	3.12 (s, 6H, CH_3_); 6.86-8.12 (m, 10H, vinyl and ArH)	292 (M^+^, 10), 105 (100), 77 (70)
**3g**	1800, 1660	2.35 (s, 3H, CH_3_); 7.07 (s, 1H, vinyl); 7.25-8.12 (m, 9H, ArH)	263 (M^+^, 10), 105 (100), 77 (70)
**3h**	1790, 1660	6.66 (q, 1H, 2-furyl);7.17-8.28 (m, 8H, vinyl , furyl and ArH)	239 (M^+^, 5), 105 (100), 77 (70)
**3i**	1810, 1660	6.89 (q, 1H, 2-thienyl);7.37-8.04 (m, 8H, vinyl, thienyl and ArH)	255 (M+, 5), 105 (100), 77 (70 )
**3j**	1780, 1650	1.34-2.13 (m, 10H, cyclohexyl); 7.34-7.88 (m, 5H, ArH)	241 (M+, 10), 105 (100), 77 (70)

## Experimental

### General

Melting points were determined on a Gallenkamp melting point apparatus and are uncorrected. Mass spectra were obtained on a Shimadzu QP 1100 EX. IR spectra were recorded with a Mattson 1000 FT-IR spectrophotometer. Nuclear magnetic resonance spectra were recorded on a Bruker DRX-500 AVANCE spectrometer using tetramethylsilane (TMS) as an internal standard. All the reactions were carried out in an unmodified domestic microwave oven BC380W having a maximum output of 900 W, operating at 2450 MHz.

### General Procedure for Synthesis of 2-phenyl-5(4H)-oxazolone derivatives **3a-j**

The appropriate aldehyde or ketone (**1**, 0.01 mol), hippuric acid (**2**, 0.01 mol), acetic anhydride (1 mL) and the appropriate catalyst (0.2 g) were introduced into a beaker and mixed. The paste thus obtained was irradiated in a microwave oven at a power output of 300 W for an appropriate time (Table 1) until the mixture had gone from white to a deep yellow, semi-solid mass. The mixture was then cooled to room temperature and was washed with cold water, and then the crude product was recrystallized from 96% ethanol (using active carbon for ruthenium chloride).
